# Impact of Intraoperative MRI on Outcomes in Pituitary Adenoma Surgery: A Systematic Review and Meta-Analysis

**DOI:** 10.7759/cureus.104926

**Published:** 2026-03-09

**Authors:** Alan M Solís Velázquez, Ana P Ruiz Lievano, Monserrat López Álvarez, Jhonatan F Jiménez Aguilar

**Affiliations:** 1 Faculty of Human Medicine "Dr. Manuel Velasco Suárez" C-II, Autonomous University of Chiapas, Tuxtla Gutiérrez, MEX; 2 Mission:Brain Chapter, Autonomous University of Chiapas, Tuxtla Gutiérrez, MEX; 3 American Association of Neurological Surgeons Chapter, Autonomous University of Chiapas, Tuxtla Gutiérrez, MEX

**Keywords:** brain tumor, intraoperative mri, neuro-oncology, neurosurgery technique, pituitary adenoma, transsphenoidal neurosurgery

## Abstract

The role of intraoperative magnetic resonance imaging (iMRI) in the surgical management of pituitary neuroendocrine tumors (PitNETs) remains debated. While transsphenoidal surgery (TSS) is the gold standard for adenoma resection, the potential benefits of iMRI in improving surgical outcomes have been increasingly recognized. This meta-analysis compares the outcomes of pituitary adenoma (PA) surgeries performed with and without iMRI, focusing on resection rates and complications in 824 patients. A comprehensive database search was conducted until January 6, 2026, to identify studies comparing surgical outcomes in patients with PA who underwent surgery with and without iMRI. The primary outcomes included gross total resection (GTR), the need for additional resection, intraoperative cerebrospinal fluid (CSF) fistulas, postoperative CSF leaks, meningitis, and the presence of a residual tumor within six months postoperatively. Among the 633 articles screened, four cohort studies met the inclusion criteria, encompassing 824 patients with PA. Patients who underwent iMRI had a significantly higher final GTR rate than those who did not (relative risk (RR) 1.71; 95% CI 1.07-2.73; P=0.03). Additionally, the residual tumor rate within six months postoperatively was significantly lower in the iMRI group (RR 0.53; 95% CI 0.35-0.80; P=0.002). Additional resection rates were comparable, with no statistically significant difference (RR 1.99; 95% CI 0.40-9.97; P=0.40). Intraoperative CSF leak rates showed no significant differences between groups (RR 1.00; 95% CI 0.29-3.48; P=1.00). Similarly, postoperative CSF leak rates were comparable (RR 0.81; 95% CI 0.29-2.25; P=0.69), and meningitis rates did not differ significantly (RR 0.62; 95% CI 0.14-2.71; P=0.53). Patients undergoing iMRI-assisted PA surgery achieve significantly higher rates of GTR and lower residual tumors at six months, without increased risk of CSF leaks, meningitis, or other complications. Further studies should assess cost-effectiveness and refine patient selection to maximize the benefits of iMRI.

## Introduction and background

Intraoperative magnetic resonance imaging (iMRI) as a tool in neurosurgery began in 1994 in Boston at Brigham and Women's Hospital [1] in collaboration with General Electric Medical Systems engineers [2]. Its implementation responded to the need to guide resection and improve technical precision, in turn providing the medical-surgical team with a real-time image that serves as a reference for the precise location of tumor remnants that would otherwise only be detectable in the postoperative period [3]. The application of these devices is based on the theoretical premise that this intervention will provide better results in terms of tumor remnants, the extent of resection, and their identification, as well as facilitating navigation and precision in their removal [4]. 

Currently, this tool, which is included in the surgical environment, has been extended for use in pituitary neuroendocrine tumors (PitNETs). These intracranial neoplasms, located in the pituitary gland, are a group of lesions that present with variable clinical expression and frequency, influenced by factors such as age, sex, and underlying comorbidities [5]. The pituitary gland, located at the skull base, controls vital endocrine functions. Each hormone produced here has a different cell line and can result in a variety of tumors [6]. Clinically, these tumors are classified as functional when they hypersecrete hormones leading to endocrine syndromes or non-functional, which primarily present with symptoms related to mass effect and compression of adjacent structures. These tumors are the third most frequent primary brain neoplasm. They account for 10% to 15% of all primary brain tumors. The prevalence of PitNETs is one in 1,000 individuals [7]. The treatment of choice for adenomas has been surgical resection [3]. Conventional transsphenoidal surgery (TSS) remains the gold standard, aiming for gross total resection (GTR), the complete removal of all visible tumors. Despite its efficacy, traditional approaches are limited by the difficulty of distinguishing pathological tissue from the normal gland in a restricted surgical field. The goal of this intervention is to achieve maximum tumor resection to normalize the patient's symptoms. These symptoms range from neurohormonal alterations that cause hyperthyroidism, acromegaly/gigantism, hypogonadism, etc., to compression symptoms. There is also the possibility of a tumor that is not symptomatic but has other manifestations attributed to the mass effect [8]. Another objective is to eliminate the difficulty of differentiating between the normal pituitary gland and the fibrotic scar tissue of the tumor, which has led to the erroneous removal of the normal gland, causing pituitary insufficiency [9]. 

Due to the wide spectrum of occurrences of pituitary adenoma (PA), results are sought that help improve resection, endocrinological symptoms, and symptoms caused by the mass effect. Some studies indicated that the extent of resection is one of the most important prognostic factors [10]. Based on the above, the implementation of iMRI in PA surgery evaluates the feasibility of better visualization of the tumor by the surgical team, which in turn increases the extent of resection and directly impacts total resection [11], and even when a gross total resection is not possible, iMRI can improve further resection to reduce tumor volume [12,13]. 

iMRI helps to recognize possible hidden or unexpected tumor remnants, achieve resection goals safely, maximize results involving the neurohumoral system, and contribute to the response involving the compression effects resulting from tumor expansion [3,4]. In addition to this, the heterogeneity in tumor size, subtypes, and growth patterns further complicates complete resection. These challenges improve the need for reliable intraoperative detection methods to guide resection [6]. Employing iMRI, the surgeon can increase the extent of resection during the index procedure itself. Considering that the residual tumor is the most important predictor of progression-free survival, the use of iMRI evidences an increase in the extent of resection [2]. However, not everything is an obvious advantage over the use of iMRI. The World Health Organization estimates 70% of medical equipment from some developed countries is unusable due to a lack of trained personnel, limitations with infrastructure, and a lack of spare parts or support for equipment [10]. A study was conducted in Germany that mentioned that most of the hospitals studied do not have iMRI (n=54, 90%), which is consistent with other European countries, where only 10% report using it [14]. It has been observed that surgery time is usually longer, in addition to representing a high cost of use, adding false-positive results and complications of installation within the surgical environment [8,12]. 

The cost-benefit ratio of the intervention must be considered, in addition to assessing the efficiency and effectiveness of the procedure. Further studies are needed to verify this relationship and prove a real benefit and to explore the added value of iMRI in pituitary surgery beyond GTR while clarifying the criteria for selecting cases that may benefit from its use [15]. While iMRI offers theoretical advantages, existing literature presents conflicting evidence regarding its actual impact on safety and long-term clinical outcomes. This lack of consensus, combined with the high costs and logistical challenges of the technology, justifies a systematic evaluation of current data. Therefore, the objective of this meta-analysis was to compare the results of PA surgeries performed with and without iMRI, focusing on resection rates and complications in 824 patients. 

## Review

Materials and methods

Study Design and Registration

This systematic review and meta-analysis was conducted in accordance with the Preferred Reporting Items for Systematic Reviews and Meta-Analyses (PRISMA) guidelines [16]. The study protocol was prospectively registered in the International Prospective Register of Systematic Reviews (PROSPERO) (registration number CRD420251087665) on July 5, 2025. No protocol deviations occurred after PROSPERO registration.

Search Strategy

A comprehensive systematic literature search was conducted in PubMed, Medical Literature Analysis and Retrieval System Online (MEDLINE), Scopus, and the Cochrane Library from database inception to January 6, 2026. No language restrictions were applied during the search process. The search strategy combined controlled vocabulary and free-text terms related to PitNETs and intraoperative imaging. Keywords included terms for PitNETs (“pituitary adenoma,” “pituitary tumor,” “PitNET,” or “hypophysis adenoma”), intraoperative magnetic resonance imaging (“intraoperative MRI,” “iMRI,” or “intraoperative magnetic resonance imaging”), and surgical approaches (“transsphenoidal surgery,” “endonasal surgery,” “hypophysectomy,” or “neurosurgery”). Search strategies were adapted to the syntax and indexing of each database to maximize sensitivity. The full search strings used for all databases (PubMed, Scopus, and Cochrane Library) are provided in Table 1.

**Table 1:** Complete search strategies used across databases Detailed search queries used for the systematic review in PubMed, Cochrane Controlled Register of Trials (CENTRAL), Scopus, and Medical Literature Analysis and Retrieval System Online (MEDLINE); Search terms combined MeSH and free-text keywords related to pituitary adenomas, intraoperative MRI, and transsphenoidal surgery. Filters and Boolean operators were adapted to each database's structure. The number of records retrieved from each database is indicated.

Full Search Phrases Used for the Respective Databases
PubMed: 399 Articles
((("intraop"[All Fields] OR "intraoperative"[All Fields] OR "intraoperatively"[All Fields]) AND ("magnetic resonance imaging"[MeSH Terms] OR ("magnetic"[All Fields] AND "resonance"[All Fields] AND "imaging"[All Fields]) OR "magnetic resonance imaging"[All Fields] OR "mri"[All Fields])) OR (("surgical procedures, operative"[MeSH Terms] OR ("surgical"[All Fields] AND "procedures"[All Fields] AND "operative"[All Fields]) OR "operative surgical procedures"[All Fields] OR "surgical"[All Fields] OR "surgically"[All Fields] OR "surgicals"[All Fields]) AND ("magnetic resonance imaging"[MeSH Terms] OR ("magnetic"[All Fields] AND "resonance"[All Fields] AND "imaging"[All Fields]) OR "magnetic resonance imaging"[All Fields] OR "mri"[All Fields])) OR ("In-surgery"[All Fields] AND ("magnetic resonance imaging"[MeSH Terms] OR ("magnetic"[All Fields] AND "resonance"[All Fields] AND "imaging"[All Fields]) OR "magnetic resonance imaging"[All Fields] OR "mri"[All Fields])) OR ("Real-time"[All Fields] AND ("magnetic resonance imaging"[MeSH Terms] OR ("magnetic"[All Fields] AND "resonance"[All Fields] AND "imaging"[All Fields]) OR "magnetic resonance imaging"[All Fields] OR "mri"[All Fields])) OR ("Intra-surgical"[All Fields] AND ("magnetic resonance imaging"[MeSH Terms] OR ("magnetic"[All Fields] AND "resonance"[All Fields] AND "imaging"[All Fields]) OR "magnetic resonance imaging"[All Fields] OR "mri"[All Fields])) OR (("operability"[All Fields] OR "operable"[All Fields] OR "operate"[All Fields] OR "operated"[All Fields] OR "operates"[All Fields] OR "operating"[All Fields] OR "operation s"[All Fields] OR "operational"[All Fields] OR "operative"[All Fields] OR "operatively"[All Fields] OR "operatives"[All Fields] OR "operator"[All Fields] OR "operator s"[All Fields] OR "operators"[All Fields] OR "surgery"[MeSH Subheading] OR "surgery"[All Fields] OR "operations"[All Fields] OR "surgical procedures, operative"[MeSH Terms] OR ("surgical"[All Fields] AND "procedures"[All Fields] AND "operative"[All Fields]) OR "operative surgical procedures"[All Fields] OR "operation"[All Fields]) AND ("magnetic resonance imaging"[MeSH Terms] OR ("magnetic"[All Fields] AND "resonance"[All Fields] AND "imaging"[All Fields]) OR "magnetic resonance imaging"[All Fields] OR "mri"[All Fields])) OR ("perioperative"[All Fields] OR "perioperatively"[All Fields]) OR (("magnetic resonance imaging"[MeSH Terms] OR ("magnetic"[All Fields] AND "resonance"[All Fields] AND "imaging"[All Fields]) OR "magnetic resonance imaging"[All Fields] OR "mri"[All Fields]) AND "Real-time"[All Fields] AND ("image"[All Fields] OR "image s"[All Fields] OR "imaged"[All Fields] OR "imager"[All Fields] OR "imager s"[All Fields] OR "imagers"[All Fields] OR "images"[All Fields] OR "imaging"[All Fields] OR "imaging s"[All Fields] OR "imagings"[All Fields])) OR ("magnetic resonance imaging"[MeSH Terms] OR ("magnetic"[All Fields] AND "resonance"[All Fields] AND "imaging"[All Fields]) OR "magnetic resonance imaging"[All Fields] OR "mri"[All Fields]) OR ("MRI-guided"[All Fields] AND ("surgery"[MeSH Subheading] OR "surgery"[All Fields] OR "surgical procedures, operative"[MeSH Terms] OR ("surgical"[All Fields] AND "procedures"[All Fields] AND "operative"[All Fields]) OR "operative surgical procedures"[All Fields] OR "general surgery"[MeSH Terms] OR ("general"[All Fields] AND "surgery"[All Fields]) OR "general surgery"[All Fields] OR "surgery s"[All Fields] OR "surgerys"[All Fields] OR "surgeries"[All Fields]) AND "In-procedure"[All Fields] AND ("magnetic resonance imaging"[MeSH Terms] OR ("magnetic"[All Fields] AND "resonance"[All Fields] AND "imaging"[All Fields]) OR "magnetic resonance imaging"[All Fields] OR "mri"[All Fields])) OR ("magneti*"[All Fields] AND "resonan*"[All Fields])) AND (((("endoscope s"[All Fields] OR "endoscoped"[All Fields] OR "endoscopes"[MeSH Terms] OR "endoscopes"[All Fields] OR "endoscope"[All Fields] OR "endoscopical"[All Fields] OR "endoscopically"[All Fields] OR "endoscopy"[MeSH Terms] OR "endoscopy"[All Fields] OR "endoscopic"[All Fields]) AND ("transsphenoid"[All Fields] OR "transsphenoidal"[All Fields] OR "transsphenoidally"[All Fields]) AND ("surgeon s"[All Fields] OR "surgeons"[MeSH Terms] OR "surgeons"[All Fields] OR "surgeon"[All Fields])) OR (("transsphenoid"[All Fields] OR "transsphenoidal"[All Fields] OR "transsphenoidally"[All Fields]) AND ("endoscope s"[All Fields] OR "endoscoped"[All Fields] OR "endoscopes"[MeSH Terms] OR "endoscopes"[All Fields] OR "endoscope"[All Fields] OR "endoscopical"[All Fields] OR "endoscopically"[All Fields] OR "endoscopy"[MeSH Terms] OR "endoscopy"[All Fields] OR "endoscopic"[All Fields]) AND ("specialist s"[All Fields] OR "specialistic"[All Fields] OR "specialization"[MeSH Terms] OR "specialization"[All Fields] OR "specialist"[All Fields] OR "specialists"[All Fields])) OR (("endonasal"[All Fields] OR "endonasally"[All Fields]) AND ("surgery"[MeSH Subheading] OR "surgery"[All Fields] OR "surgical procedures, operative"[MeSH Terms] OR ("surgical"[All Fields] AND "procedures"[All Fields] AND "operative"[All Fields]) OR "operative surgical procedures"[All Fields] OR "general surgery"[MeSH Terms] OR ("general"[All Fields] AND "surgery"[All Fields]) OR "general surgery"[All Fields] OR "surgery s"[All Fields] OR "surgerys"[All Fields] OR "surgeries"[All Fields]) AND ("practitioner"[All Fields] OR "practitioner s"[All Fields] OR "practitioners"[All Fields])) OR ("Minimally"[All Fields] AND ("invasibility"[All Fields] OR "invasible"[All Fields] OR "invasion"[All Fields] OR "invasions"[All Fields] OR "invasive"[All Fields] OR "invasively"[All Fields] OR "invasiveness"[All Fields] OR "invasives"[All Fields] OR "invasivity"[All Fields]) AND ("skull base"[MeSH Terms] OR ("skull"[All Fields] AND "base"[All Fields]) OR "skull base"[All Fields]) AND ("surgeon s"[All Fields] OR "surgeons"[MeSH Terms] OR "surgeons"[All Fields] OR "surgeon"[All Fields])) OR (("transsphenoid"[All Fields] OR "transsphenoidal"[All Fields] OR "transsphenoidally"[All Fields]) AND ("approach"[All Fields] OR "approach s"[All Fields] OR "approachability"[All Fields] OR "approachable"[All Fields] OR "approache"[All Fields] OR "approached"[All Fields] OR "approaches"[All Fields] OR "approaching"[All Fields] OR "approachs"[All Fields]) AND ("specialist s"[All Fields] OR "specialistic"[All Fields] OR "specialization"[MeSH Terms] OR "specialization"[All Fields] OR "specialist"[All Fields] OR "specialists"[All Fields])) OR (("endoscope s"[All Fields] OR "endoscoped"[All Fields] OR "endoscopes"[MeSH Terms] OR "endoscopes"[All Fields] OR "endoscope"[All Fields] OR "endoscopical"[All Fields] OR "endoscopically"[All Fields] OR "endoscopy"[MeSH Terms] OR "endoscopy"[All Fields] OR "endoscopic"[All Fields]) AND ("skull base"[MeSH Terms] OR ("skull"[All Fields] AND "base"[All Fields]) OR "skull base"[All Fields]) AND ("surgeon s"[All Fields] OR "surgeons"[MeSH Terms] OR "surgeons"[All Fields] OR "surgeon"[All Fields])) OR (("endonasal"[All Fields] OR "endonasally"[All Fields]) AND ("endoscope s"[All Fields] OR "endoscoped"[All Fields] OR "endoscopes"[MeSH Terms] OR "endoscopes"[All Fields] OR "endoscope"[All Fields] OR "endoscopical"[All Fields] OR "endoscopically"[All Fields] OR "endoscopy"[MeSH Terms] OR "endoscopy"[All Fields] OR "endoscopic"[All Fields]) AND ("surgeon s"[All Fields] OR "surgeons"[MeSH Terms] OR "surgeons"[All Fields] OR "surgeon"[All Fields])) OR (("transnasal"[All Fields] OR "transnasally"[All Fields]) AND ("surgery"[MeSH Subheading] OR "surgery"[All Fields] OR "surgical procedures, operative"[MeSH Terms] OR ("surgical"[All Fields] AND "procedures"[All Fields] AND "operative"[All Fields]) OR "operative surgical procedures"[All Fields] OR "general surgery"[MeSH Terms] OR ("general"[All Fields] AND "surgery"[All Fields]) OR "general surgery"[All Fields] OR "surgery s"[All Fields] OR "surgerys"[All Fields] OR "surgeries"[All Fields]) AND ("expert"[All Fields] OR "expert s"[All Fields] OR "expertize"[All Fields] OR "experts"[All Fields])) OR (("skull base"[MeSH Terms] OR ("skull"[All Fields] AND "base"[All Fields]) OR "skull base"[All Fields]) AND ("endoscope s"[All Fields] OR "endoscoped"[All Fields] OR "endoscopes"[MeSH Terms] OR "endoscopes"[All Fields] OR "endoscope"[All Fields] OR "endoscopical"[All Fields] OR "endoscopically"[All Fields] OR "endoscopy"[MeSH Terms] OR "endoscopy"[All Fields] OR "endoscopic"[All Fields]) AND ("specialist s"[All Fields] OR "specialistic"[All Fields] OR "specialization"[MeSH Terms] OR "specialization"[All Fields] OR "specialist"[All Fields] OR "specialists"[All Fields])) OR (("transsphenoid"[All Fields] OR "transsphenoidal"[All Fields] OR "transsphenoidally"[All Fields]) AND ("methods"[MeSH Terms] OR "methods"[All Fields] OR "procedure"[All Fields] OR "methods"[MeSH Subheading] OR "procedures"[All Fields] OR "procedural"[All Fields] OR "procedurally"[All Fields] OR "procedure s"[All Fields]) AND ("operability"[All Fields] OR "operable"[All Fields] OR "operate"[All Fields] OR "operated"[All Fields] OR "operates"[All Fields] OR "operating"[All Fields] OR "operation s"[All Fields] OR "operational"[All Fields] OR "operative"[All Fields] OR "operatively"[All Fields] OR "operatives"[All Fields] OR "operator"[All Fields] OR "operator s"[All Fields] OR "operators"[All Fields] OR "surgery"[MeSH Subheading] OR "surgery"[All Fields] OR "operations"[All Fields] OR "surgical procedures, operative"[MeSH Terms] OR ("surgical"[All Fields] AND "procedures"[All Fields] AND "operative"[All Fields]) OR "operative surgical procedures"[All Fields] OR "operation"[All Fields])) OR "ETSS"[All Fields]) AND ((("transsphenoid"[All Fields] OR "transsphenoidal"[All Fields] OR "transsphenoidally"[All Fields]) AND ("microsurgery"[MeSH Terms] OR "microsurgery"[All Fields] OR "microsurgeries"[All Fields])) OR (("microscop"[All Fields] OR "microscopal"[All Fields] OR "microscope"[All Fields] OR "microscope s"[All Fields] OR "microscopes"[All Fields] OR "microscopic"[All Fields] OR "microscopical"[All Fields] OR "microscopically"[All Fields] OR "microscopics"[All Fields]) AND ("transsphenoid"[All Fields] OR "transsphenoidal"[All Fields] OR "transsphenoidally"[All Fields]) AND ("surgery"[MeSH Subheading] OR "surgery"[All Fields] OR "surgical procedures, operative"[MeSH Terms] OR ("surgical"[All Fields] AND "procedures"[All Fields] AND "operative"[All Fields]) OR "operative surgical procedures"[All Fields] OR "general surgery"[MeSH Terms] OR ("general"[All Fields] AND "surgery"[All Fields]) OR "general surgery"[All Fields] OR "surgery s"[All Fields] OR "surgerys"[All Fields] OR "surgeries"[All Fields])) OR (("microsurgical"[All Fields] OR "microsurgically"[All Fields]) AND ("transsphenoid"[All Fields] OR "transsphenoidal"[All Fields] OR "transsphenoidally"[All Fields]) AND ("approach"[All Fields] OR "approach s"[All Fields] OR "approachability"[All Fields] OR "approachable"[All Fields] OR "approache"[All Fields] OR "approached"[All Fields] OR "approaches"[All Fields] OR "approaching"[All Fields] OR "approachs"[All Fields])) OR (("microscop"[All Fields] OR "microscopal"[All Fields] OR "microscope"[All Fields] OR "microscope s"[All Fields] OR "microscopes"[All Fields] OR "microscopic"[All Fields] OR "microscopical"[All Fields] OR "microscopically"[All Fields] OR "microscopics"[All Fields]) AND ("skull base surg"[Journal] OR ("skull"[All Fields] AND "base"[All Fields] AND "surgery"[All Fields]) OR "skull base surgery"[All Fields])) OR (("transnasal"[All Fields] OR "transnasally"[All Fields]) AND ("microsurgery"[MeSH Terms] OR "microsurgery"[All Fields] OR "microsurgeries"[All Fields])) OR (("microscop"[All Fields] OR "microscopal"[All Fields] OR "microscope"[All Fields] OR "microscope s"[All Fields] OR "microscopes"[All Fields] OR "microscopic"[All Fields] OR "microscopical"[All Fields] OR "microscopically"[All Fields] OR "microscopics"[All Fields]) AND ("pituitaries"[All Fields] OR "pituitary gland"[MeSH Terms] OR ("pituitary"[All Fields] AND "gland"[All Fields]) OR "pituitary gland"[All Fields] OR "pituitary"[All Fields] OR "pituitary s"[All Fields]) AND ("surgery"[MeSH Subheading] OR "surgery"[All Fields] OR "surgical procedures, operative"[MeSH Terms] OR ("surgical"[All Fields] AND "procedures"[All Fields] AND "operative"[All Fields]) OR "operative surgical procedures"[All Fields] OR "general surgery"[MeSH Terms] OR ("general"[All Fields] AND "surgery"[All Fields]) OR "general surgery"[All Fields] OR "surgery s"[All Fields] OR "surgerys"[All Fields] OR "surgeries"[All Fields])) OR (("microsurgical"[All Fields] OR "microsurgically"[All Fields]) AND ("transnasal"[All Fields] OR "transnasally"[All Fields]) AND ("approach"[All Fields] OR "approach s"[All Fields] OR "approachability"[All Fields] OR "approachable"[All Fields] OR "approache"[All Fields] OR "approached"[All Fields] OR "approaches"[All Fields] OR "approaching"[All Fields] OR "approachs"[All Fields])) OR (("transsphenoid"[All Fields] OR "transsphenoidal"[All Fields] OR "transsphenoidally"[All Fields]) AND ("microsurgery"[MeSH Terms] OR "microsurgery"[All Fields] OR ("microsurgical"[All Fields] AND "procedure"[All Fields]) OR "microsurgical procedure"[All Fields])) OR (("microsurgical"[All Fields] OR "microsurgically"[All Fields]) AND ("pituitaries"[All Fields] OR "pituitary gland"[MeSH Terms] OR ("pituitary"[All Fields] AND "gland"[All Fields]) OR "pituitary gland"[All Fields] OR "pituitary"[All Fields] OR "pituitary s"[All Fields]) AND ("removability"[All Fields] OR "removal"[All Fields] OR "removals"[All Fields] OR "remove"[All Fields] OR "removed"[All Fields] OR "removement"[All Fields] OR "remover"[All Fields] OR "removers"[All Fields] OR "removes"[All Fields] OR "removing"[All Fields])) OR "MTSS"[All Fields])) AND ("pituitary neoplasms"[MeSH Terms] OR ("pituitary"[All Fields] AND "neoplasms"[All Fields]) OR "pituitary neoplasms"[All Fields] OR ("pituitary"[All Fields] AND "adenomas"[All Fields]) OR "pituitary adenomas"[All Fields] OR ("pituitary tumours"[All Fields] OR "pituitary neoplasms"[MeSH Terms] OR ("pituitary"[All Fields] AND "neoplasms"[All Fields]) OR "pituitary neoplasms"[All Fields] OR ("pituitary"[All Fields] AND "tumors"[All Fields]) OR "pituitary tumors"[All Fields]) OR (("pituitary gland"[MeSH Terms] OR ("pituitary"[All Fields] AND "gland"[All Fields]) OR "pituitary gland"[All Fields] OR "hypophyseal"[All Fields]) AND ("adenoma"[MeSH Terms] OR "adenoma"[All Fields] OR "adenomas"[All Fields] OR "adenoma s"[All Fields])) OR (("pituitary gland"[MeSH Terms] OR ("pituitary"[All Fields] AND "gland"[All Fields]) OR "pituitary gland"[All Fields]) AND ("neoplasm s"[All Fields] OR "neoplasms"[MeSH Terms] OR "neoplasms"[All Fields] OR "neoplasm"[All Fields])) OR (("hypophysial"[All Fields] OR "pituitary gland"[MeSH Terms] OR ("pituitary"[All Fields] AND "gland"[All Fields]) OR "pituitary gland"[All Fields] OR "hypophysis"[All Fields]) AND ("adenoma"[MeSH Terms] OR "adenoma"[All Fields] OR "adenomas"[All Fields] OR "adenoma s"[All Fields])) OR (("pituitaries"[All Fields] OR "pituitary gland"[MeSH Terms] OR ("pituitary"[All Fields] AND "gland"[All Fields]) OR "pituitary gland"[All Fields] OR "pituitary"[All Fields] OR "pituitary s"[All Fields]) AND ("lesion"[All Fields] OR "lesion s"[All Fields] OR "lesional"[All Fields] OR "lesions"[All Fields])) OR ("pituitary neoplasms"[MeSH Terms] OR ("pituitary"[All Fields] AND "neoplasms"[All Fields]) OR "pituitary neoplasms"[All Fields] OR ("pituitary"[All Fields] AND "gland"[All Fields] AND "tumors"[All Fields]) OR "pituitary gland tumors"[All Fields]) OR (("pituitaries"[All Fields] OR "pituitary gland"[MeSH Terms] OR ("pituitary"[All Fields] AND "gland"[All Fields]) OR "pituitary gland"[All Fields] OR "pituitary"[All Fields] OR "pituitary s"[All Fields]) AND ("growth and development"[MeSH Subheading] OR ("growth"[All Fields] AND "development"[All Fields]) OR "growth and development"[All Fields] OR "growth"[All Fields] OR "growth"[MeSH Terms] OR "growths"[All Fields])) OR (("hypophysial"[All Fields] OR "pituitary gland"[MeSH Terms] OR ("pituitary"[All Fields] AND "gland"[All Fields]) OR "pituitary gland"[All Fields] OR "hypophysis"[All Fields]) AND ("neoplasm s"[All Fields] OR "neoplasms"[MeSH Terms] OR "neoplasms"[All Fields] OR "neoplasm"[All Fields])) OR (("sellar"[All Fields] OR "sellars"[All Fields]) AND ("cysts"[MeSH Terms] OR "cysts"[All Fields] OR "cyst"[All Fields] OR "neurofibroma"[MeSH Terms] OR "neurofibroma"[All Fields] OR "neurofibromas"[All Fields] OR "tumor s"[All Fields] OR "tumoral"[All Fields] OR "tumorous"[All Fields] OR "tumour"[All Fields] OR "neoplasms"[MeSH Terms] OR "neoplasms"[All Fields] OR "tumor"[All Fields] OR "tumour s"[All Fields] OR "tumoural"[All Fields] OR "tumourous"[All Fields] OR "tumours"[All Fields] OR "tumors"[All Fields])))
Cochrane Controlled Register of Trials (CENTRAL): 8 Articles
1 Pituitary adenoma
2 hypophysis
3 adenoma
4 macroadenoma
5 macro-adenoma
6 microadenoma
7 micro-adenoma
8 #1 or #2
9 #3 or #4 or #5 or #6 or #7
10 #8 and #9
11 MeSH descriptor: Pituitary neoplasms explode all trees
12 #10 or #11
13 intraoperative MRI
14 intraop*
15 intra-op*
16 #13 or #14
17 magnetic resonance imag*
18 MRI
19 #16 or #17 or #18
20 #15 and #19
21 transsphenoidal surgery
22 transsphenoidal
23 trans-sphenoidal
24 endonasal
25 endo-nasal
26 #21 or #22 or #23 or #24
27 resect*
28 surg*
29 hypophysectomy
30 Any MeSH descriptor in all MeSH products
31 MeSH descriptor: Neurosurgery explode all trees
32 #26 or #27 or #28 or #29 or #30
33 #25 or #31
34 #12 and #20 and #32
Scopus: 227 Articles
Intraoperative AND magnetic AND resonance AND imaging AND transsphenoidal AND resection AND pituitary AND adenoma
Medline: 27 Articles
("pituitary neoplasms"[MeSH Terms] OR Pituitary tumor[Text Word]) OR (("pituitary gland"[MeSH Terms] OR Hypophyseal[Text Word]) AND ("adenoma"[MeSH Terms] OR adenoma[Text Word])) OR "pituitary neoplasms"[MeSH Terms] OR ("pituitary gland"[MeSH Terms] OR Pituitary gland[Text Word]) OR (("pituitary gland"[MeSH Terms] OR Pituitary[Text Word]) AND ("human body"[MeSH Terms] OR body[Text Word]) AND ("adenoma"[MeSH Terms] OR Adenoma[Text Word]) AND ("neoplasms"[MeSH Terms] OR Benign tumor[Text Word])) OR (Glandular[All Fields] AND ("neoplasms"[MeSH Terms] OR tumor[Text Word])) OR (Non-cancerous[All Fields] AND ("neoplasms"[MeSH Terms] OR neoplasm[Text Word])) OR (Large[All Fields] AND ("pituitary neoplasms"[MeSH Terms] OR pituitary adenoma[Text Word])) OR (Non-functioning[All Fields] AND ("adenoma"[MeSH Terms] OR adenoma[Text Word])) OR (Small[All Fields] AND ("pituitary neoplasms"[MeSH Terms] OR pituitary adenoma[Text Word]) AND ("pituitary neoplasms"[MeSH Terms] OR Pituitary neoplasms[Text Word])) OR ("pituitary neoplasms"[MeSH Terms] OR Pituitary tumors[Text Word]) OR (("pituitary gland"[MeSH Terms] OR Hypophyseal[Text Word]) AND ("neoplasms"[MeSH Terms] OR tumors[Text Word])) OR (("pituitary gland"[MeSH Terms] OR Pituitary[Text Word]) AND lesions[All Fields] AND Intraoperative[All Fields] AND ("magnetic resonance imaging"[MeSH Terms] OR MRI[Text Word])) OR (Real-time[All Fields] AND ("magnetic resonance imaging"[MeSH Terms] OR MRI[Text Word])) OR (("surgical procedures, operative"[MeSH Terms] OR Surgical[Text Word]) AND ("magnetic resonance imaging"[MeSH Terms] OR MRI[Text Word]) AND guidance[All Fields] AND "magnetic resonance imaging"[MeSH Terms] AND "magnetic resonance imaging"[MeSH Terms] AND ("surgery"[Subheading] OR "surgical procedures, operative"[MeSH Terms] OR "general surgery"[MeSH Terms] OR surgery[Text Word])) OR (("endoscopy"[MeSH Terms] OR Endoscopic[Text Word]) AND ("surgery"[Subheading] OR "surgical procedures, operative"[MeSH Terms] OR "general surgery"[MeSH Terms] OR surgery[Text Word])) OR (("pituitary gland"[MeSH Terms] OR Pituitary[Text Word]) AND ("surgery"[Subheading] OR "surgical procedures, operative"[MeSH Terms] OR "general surgery"[MeSH Terms] OR surgery[Text Word]) AND ("sphenoid sinus"[MeSH Terms] OR sphenoid sinus[Text Word])) OR (("endoscopy"[MeSH Terms] OR Endoscopic[Text Word]) AND ("nose"[MeSH Terms] OR nasal[Text Word])) OR Transnasal[All Fields] OR ("hypophysectomy"[MeSH Terms] OR Hypophysectomy[Text Word]) OR "hypophysectomy"[MeSH Terms] OR (("surgical procedures, operative"[MeSH Terms] OR Surgical[Text Word]) AND removal[All Fields] AND ("pituitary gland"[MeSH Terms] OR pituitary[Text Word])) OR (("pituitary gland"[MeSH Terms] OR Pituitary[Text Word]) AND ablation[All Fields])

Additionally, a manual search using the snowballing technique was performed by reviewing the reference lists of all included studies and relevant systematic reviews to identify additional eligible studies not retrieved through the electronic database search.

Eligibility Criteria (Inclusion and Exclusion Criteria)

The research question was structured according to the Population, Intervention, Control, and Outcomes (PICO) framework as follows: In adult patients with PitNETs, does iMRI-assisted TSS, compared with conventional TSS without iMRI, improve GTR rates and surgical safety outcomes?

The population included adult patients diagnosed with functional or non-functional PitNETs, including invasive tumors (Knosp grades III-IV). The intervention consisted of TSS resection assisted by intraoperative MRI, while the comparator was conventional TSS without iMRI. The primary outcome was the GTR rate. Secondary outcomes included residual tumor within six months, the need for additional resections, and postoperative complications such as cerebrospinal fluid (CSF) leaks and meningitis.

Eligible studies were original, peer-reviewed observational or interventional studies that directly compared iMRI-assisted versus non-iMRI TSS in adult patients. Studies were required to include at least five participants, evaluate the extent of resection as a primary outcome, and provide sufficient detail regarding patient characteristics, surgical approach, and clinical outcomes.

We excluded non-original publications, including narrative or systematic reviews, editorials, commentaries, letters, conference abstracts, protocols, institutional reports, theses, and book chapters. Case series were excluded regardless of sample size, as they do not provide a direct comparison between iMRI-assisted and non-iMRI procedures. Animal or in vitro studies and studies focusing exclusively on craniopharyngiomas or Rathke’s cleft cysts were also excluded. Additionally, studies that did not assess the extent of resection as a primary outcome were not considered.

Study Selection

Two reviewers (A.R.L. and M.L.A.) independently screened titles and abstracts to identify potentially eligible studies. Full-text articles were subsequently reviewed to confirm eligibility based on the predefined inclusion and exclusion criteria. Any disagreements were resolved through discussion and consensus or, when necessary, by consultation with a third reviewer (A.S.V.).

Data Extraction

Data extraction was performed using a predefined standardized Microsoft Excel form (Microsoft Corporation, Redmond, WA, USA). Extracted variables included first author, year of publication, study design, sample size, patient demographics (mean age, sex distribution, and reported comorbidities), tumor characteristics (adenoma type, size, functionality, and cavernous sinus invasion), and surgical outcomes (extent of resection, intraoperative detection of residual tumor, additional resections, and six-month follow-up findings). Postoperative complications, including CSF leaks, meningitis, and transient diabetes insipidus, were also recorded.

Three investigators independently extracted the data, and a fourth reviewer verified all entries to ensure accuracy and consistency. Studies with missing or 'not reported' data for the primary outcomes were excluded from the quantitative synthesis, and no data imputation was performed to maintain the reliability of the pooled estimates. Authors of the included studies were not contacted for additional or unpublished data.

Risk of Bias Assessment

The risk of bias of the included studies was assessed using the Risk of Bias in Nonrandomized Studies of Interventions (ROBINS-I) tool [17], which evaluates seven domains: bias due to confounding, selection of participants, classification of interventions, deviations from intended interventions, missing data, measurement of outcomes, and selective reporting.

Two reviewers (A.P.R.L. and M.L.A.) independently performed the risk of bias assessment for each included study. Discrepancies were resolved through discussion and consensus with a third reviewer (A.M.S.V.).

Potential publication bias was evaluated by visual inspection of funnel plots for the primary outcome. Due to the limited number of included studies (n = 4), formal statistical tests for publication bias, such as Egger’s or Begg’s tests, were not performed because of insufficient statistical power.

Outcome Measures

Outcomes were analyzed by comparing patients undergoing iMRI-assisted TSS with those receiving conventional TSS without iMRI. The primary outcome was the GTR rate, defined as the complete absence of visible tumor on postoperative imaging. Secondary outcomes included residual tumor within six months after surgery, the need for additional resections, postoperative mortality, and surgical safety outcomes.

Safety was assessed based on the incidence of intraoperative and postoperative complications, particularly CSF leaks and meningitis.

Statistical Analysis

For dichotomous outcomes, pooled relative risks (RRs) with 95% CIs were calculated, and a p-value ≤ 0.05 was considered statistically significant. Statistical heterogeneity was assessed using Cochran’s Q test, with p < 0.10 indicating significant heterogeneity, and quantified using the I² statistic. I² values were interpreted as <40% indicating low heterogeneity, 40-75% moderate heterogeneity, and >75% high heterogeneity.

A fixed-effects model was applied when heterogeneity was low, whereas a random-effects model was used in cases of moderate or high heterogeneity. Forest plots were generated to visually present pooled effect estimates. To assess the robustness of the findings and identify potential sources of heterogeneity, a leave-one-out sensitivity analysis was performed for the primary outcome. All statistical analyses were conducted using Review Manager (RevMan) software, version 6.0 (The Cochrane Collaboration, London, UK).

Results

Characteristics of the Included Studies

**Figure 1:**
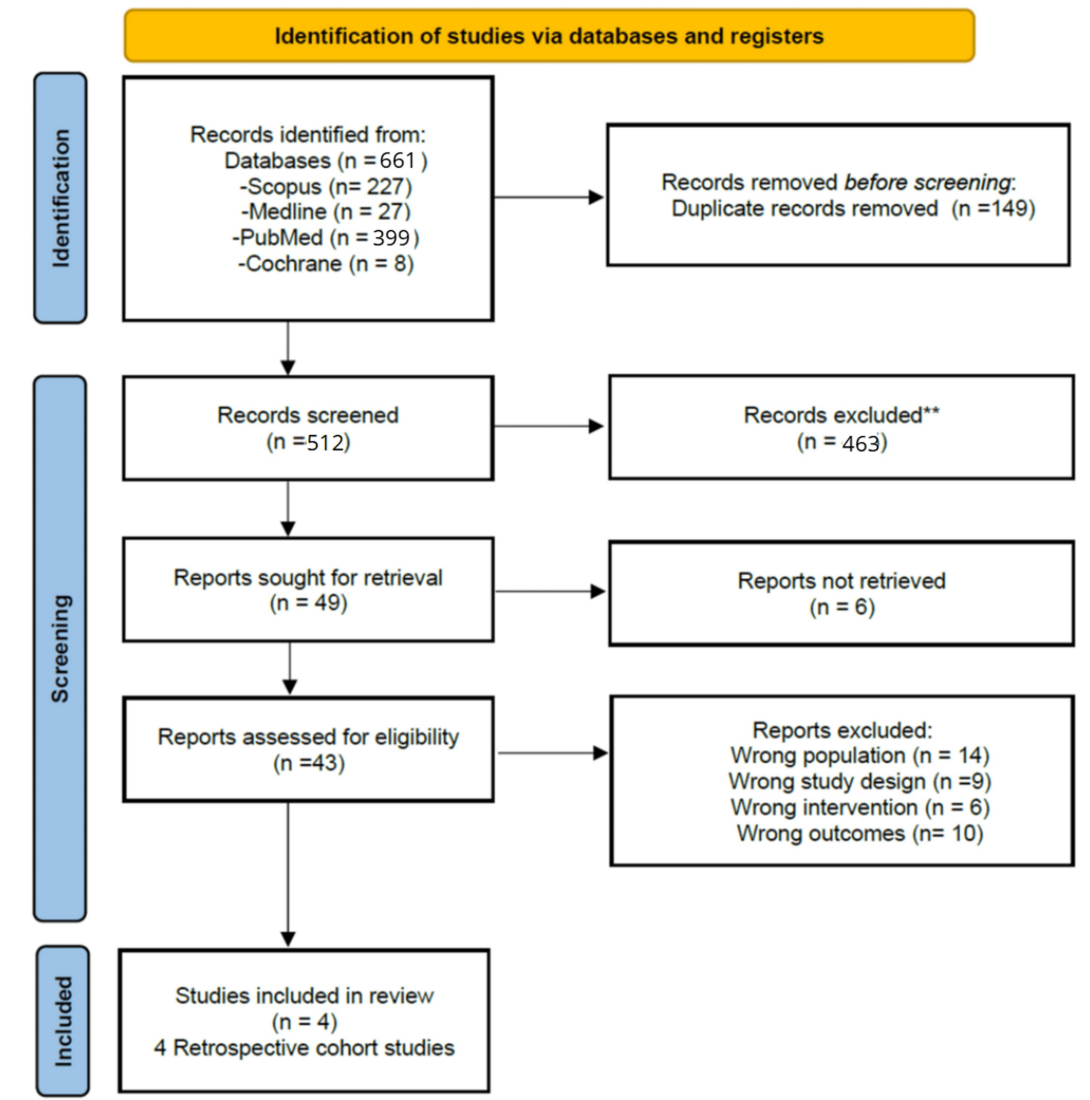
A PRISMA flowchart outlining the study selection process PRISMA: Preferred Reporting Items for Systematic Reviews and Meta-Analysis; MEDLINE: Medical Literature Analysis and Retrieval System Online

The systematic search yielded a total of 661 publications. After screening and eligibility assessment, only four retrospective cohort studies [18-21] met the inclusion criteria. These are detailed in the PRISMA 2020 flow diagram (Figure 1).

Among the selected studies, two were conducted in the United States [18,20], one in Japan [19], and one in Germany [21]. In total, these studies analyzed 824 patients who underwent transsphenoidal resection of PitNETs. Of these, 309 patients received iMRI-assisted surgery, while 515 underwent surgery without iMRI guidance. The methodological and demographic characteristics of each study, such as author, publication year, country, study design, study period, sample size, patient groups, and main findings, are summarized in Table 2.

**Table 2:** Summary of retrospective studies evaluating the role of intraoperative MRI in pituitary adenoma surgery

Author & year	Country	Study design	Study period	Population	Groups	Main findings
Patel et al., 2022 [18]	United States of America	Retrospective cohort study	2013-2021	15 patients with pituitary neuroendocrine tumors (PitNETs)	Intraoperative MRI (iMRI) group (n=87, 24.8%) vs. non-iMRI group (n=264, 75.2%)	iMRI significantly improved gross total resection (GTR) rates, especially in complex anatomical regions like the suprasellar space and cavernous sinus. The increase in GTR did not lead to higher complication rates or endocrine dysfunction. iMRI showed no false negatives and enabled safer, targeted resections. The authors conclude that iMRI adds substantial clinical and economic value to pituitary surgery.
Ogiwara et al., 2021 [19]	Japan	Retrospective cohort study	2016-2018	15 patients with acromegaly	iMRI group (n = 6, 40.0%) vs. non-iMRI group (n = 9, 60.0%)	iMRI significantly increased GTR in acromegaly surgery, but did not clearly improve hormonal remission rates. The authors suggest that relying solely on iMRI may cause surgeons to be less aggressive before imaging, possibly contributing to false-negative endocrine outcomes. They emphasize the need to combine iMRI with high-definition endoscopic visualization and surgical expertise to achieve hormonal remission.
Berkmann et al., 2012 [21]	Germany	Retrospective cohort study	2006-2010	92 patients with non-functioning PitNETs (60 iMRI, 32 control)	iMRI group (n = 60, 65.2%) vs. non-iMRI group (n = 32, 34.8%)	Use of iMRI significantly improved the rate of complete tumor removal (gross-total resection) in patients with non-functioning PitNETs, as compared to the control group without iMRI. Additionally, patients in the iMRI group showed better postoperative endocrine recovery. The study concluded that iMRI is a valuable tool for enhancing surgical success without increasing the rate of complications.
Sylvester et al.,2015 [20]	United States of America	Retrospective cohort study	1998–2012	339 patients (aged 19–79.5 years), both functional and non-functional adenomas	iMRI group (n = 156, 42.6%) vs. non-iMRI group (n = 210, 57.4%)	Combined use of iMRI and endoscopy significantly increased the extent of resection and was associated with longer progression-free survival compared to conventional transsphenoidal microsurgery. The study found no significant increase in complication rates with the combined approach. Increased resection status (gross vs. near vs. sub-total) was an independent predictor of improved clinical outcomes. iMRI alone or endoscopy alone did not yield statistically significant advantages, but their combination did.

All patients included in the analysis were diagnosed with PitNETs. Out of the total cohort, 328 cases (39.8%) were classified as functional adenomas, while 496 (60.2%) were non-functional. Specifically, within the iMRI group, 151 functional adenomas were identified. Table 3 provides a detailed overview of the clinical and morphological characteristics across the included studies. These include patient age, sex distribution, tumor size, Knosp classification, and the presence of suprasellar invasion. Additionally, the table outlines intraoperative and postoperative outcomes such as GTR rates, residual tumor observed within six months postoperatively, and surgical complications, including CSF leaks and meningitis.

**Table 3:** Demographic, tumor, surgical, and outcome characteristics of the included studies in the review iMRI: intraoperative magnetic resonance imaging; GTR: gross total resection; CSF: cerebrospinal fluid; NR: not reported; mm: millimeters

Identifiers	Demography				Tumor characteristics					Outcomes				
					Tumor features		Preoperative tumor characteristics			Postoperative outcomes		Surgical complications		
Authors	Groups	Population size	Age (years)	Sex (female/male)	Functional adenoma	Non-functional adenoma	Maximum diameter (mm)	Knosp grade	Cavernous sinus invasion	Final gross-total resection	Residual tumor six months postoperative	Intraoperative CSF fistula	Postoperative CSF leaks	Meningitis
Patel et al., 2022 [18]	iMRI	87	49.1 mean	Female: 36 Male: 51	56	239	NR	I: 154 II:81 IIIa: 52 IIIb: 15 IV: 49	33	71	16	NR	4	NR
	No iMRI	264	51.8 mean	Female: 145 Male: 119	111	NR	NR	NR	148	172	92	NR	14	NR
Ogiwara et al., 2021 [19]	iMRI	6	49.3 (38-70)	Female: 2 Male:4	15	NR	12.4 mean (range: 5.6-16.8)	I:1 II:3 IIIa:1 IIIb: NR IV: NR	NR	6	NR	1	0	0
	No iMRI	9	45.1 (9-69)	Female:5 Male:4		NR	16.7 mean (range: 9.9-23.2)	I:4 II: 2 IIIa:2 IIIb:NR IV:NR	NR	4	NR	4	1	0
Sylvester et al.,2015 [20]	No iMRI	210	48.2 (14.9 Mean)	Female: 111 Male: 99	80	130	23.3 (mean 12,6)	NR	NR	36	NR	NR	NR	4
	iMRI	156	48.6 (13.4 mean)	Female: 89 Male: 67	66	90	21.3 (mean 12.7)	NR	NR	56	NR	NR	NR	2
Berkmann et al., 2012 [21]	iMRI	60	59± 15 Mean	Female: 18 Male:42	NR	60	NR	NR	NR	51	9	17	NR	NR
	No iMRI	32	59+-14 mean	Female:12 Male:20	NR	32	NR	NR	NR	22	9	6	NR	NR

Clinical and Surgical Outcomes

**Figure 2:**
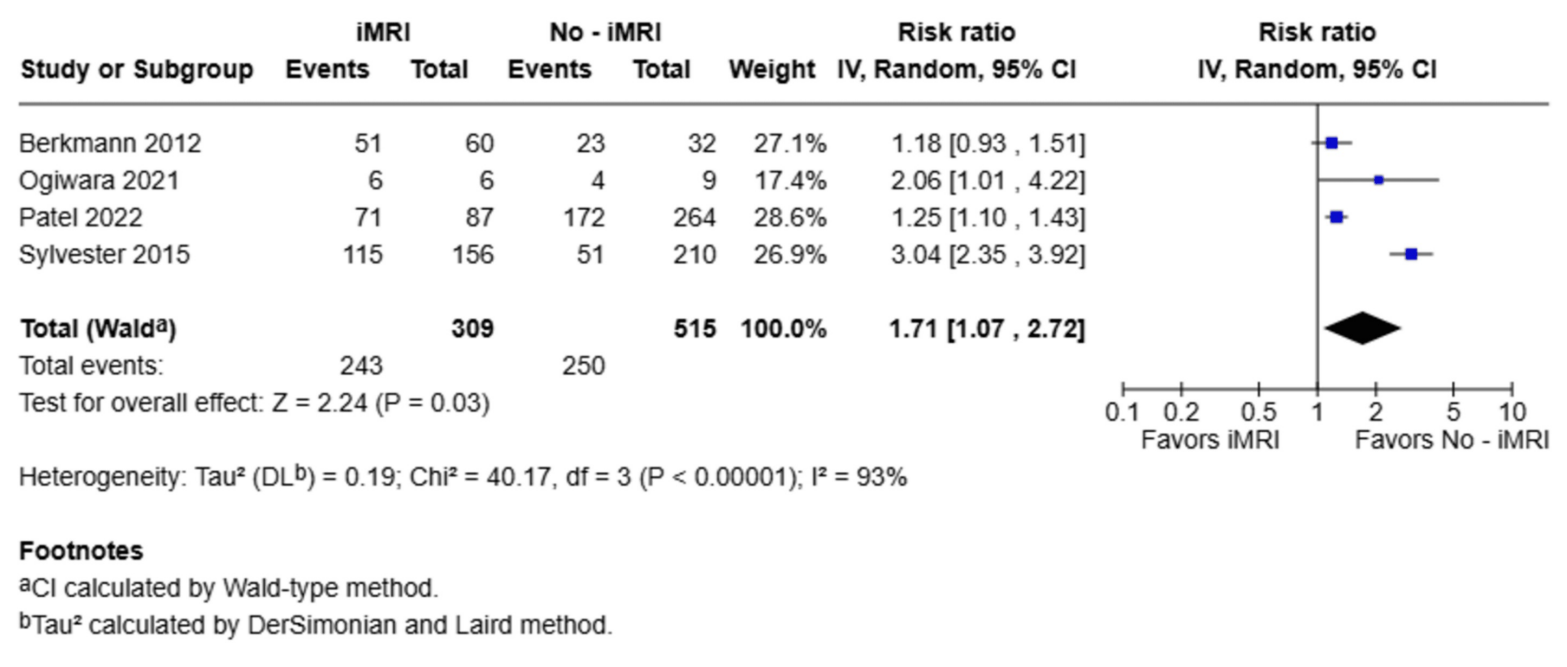
Forest plot comparing final GTR between iMRI and non-iMRI groups Studies included: Berkmann et al., 2012 [21], Ogiwara et al., 2021 [19], Patel et al., 2022 [18], Sylvester et al., 2015 [20] Forest plot displaying the RR and 95% CI for achieving final GTR in patients undergoing pituitary adenoma surgery with iMRI versus without. The pooled analysis demonstrates a statistically significant benefit in favor of the iMRI group (RR = 1.71; 95% CI: 1.07–2.72; p = 0.03). RR: risk ratio; GTR: gross total resection; iMRI: intraoperative magnetic resonance imaging

The primary outcome analyzed across the four included studies was the final GTR. Each study directly compared GTR rates between patients who underwent iMRI-assisted transsphenoidal resection and those who did not. The pooled meta-analysis revealed that the use of iMRI was significantly associated with a higher likelihood of achieving complete resection, with an RR of 1.71 (95% CI: 1.07-2.72; p = 0.03), as illustrated in Figure 2. Significant heterogeneity was observed in this initial analysis (I² = 93%). To evaluate the robustness of these findings, a leave-one-out sensitivity analysis was conducted, which revealed that this high heterogeneity was primarily driven by the study by Sylvester et al. [20]. Upon exclusion of this study, the heterogeneity decreased drastically to I² = 4%, while the benefit of iMRI remained statistically significant (RR = 1.25; 95% CI: 1.11-1.42; p = 0.0003). Although the exclusion of other individual studies (Patel [18] or Ogiwara [19]) marginally shifted the p-value above the 0.05 threshold due to the limited number of included trials, the direction of the effect consistently favored the use of iMRI across all iterations.

**Figure 3:**
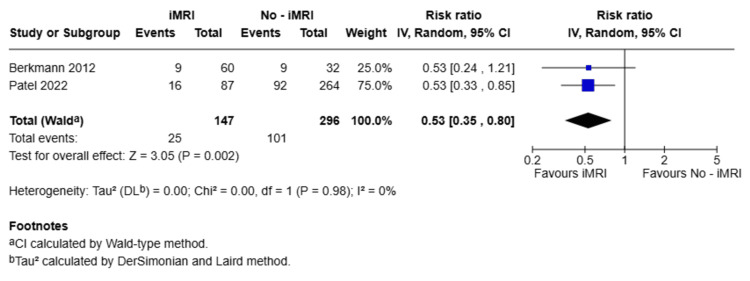
Forest plot comparing residual tumor rates within six months postoperatively between iMRI and non-iMRI groups Studies included: Berkmann et al., 2012 [21], Patel et al., 2022 [18] Forest plot illustrating the RR and 95% CI for the presence of residual tumor within six months after surgery. Patients in the iMRI group had a significantly lower likelihood of residual tumor compared to the non-iMRI group (RR = 0.53; 95% CI: 0.35-0.80; p = 0.002). RR: risk ratio; iMRI: intraoperative magnetic resonance imaging

Regarding secondary outcomes, all studies evaluated the presence of residual tumor within the first six months after surgery. Patients in the iMRI group demonstrated a significantly lower rate of residual tumor compared to the non-iMRI group, with a pooled RR of 0.53 (95%CI: 0.35-0.80; p = 0.002), as shown in Figure 3.

**Figure 4:**
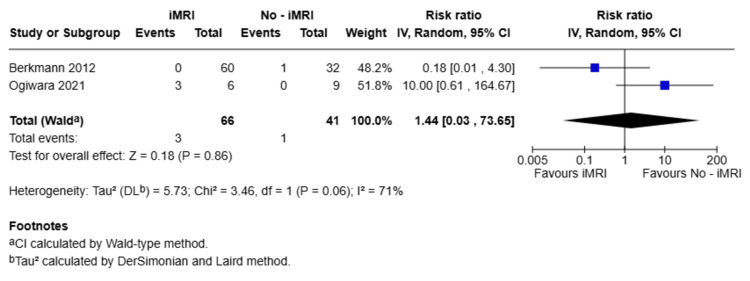
Forest plot of additional resection rates comparing iMRI and non-iMRI groups Studies included: Berkmann et al., 2012 [21], Ogiwara et al., 2021 [19] Forest plot depicting the RR and 95% CI for the need of additional surgical resection in patients undergoing pituitary adenoma surgery with or without iMRI. No statistically significant difference was observed between groups (RR = 1.44; 95% CI: 0.03-73.65; p = 0.86). RR: risk ratio; iMRI: intraoperative magnetic resonance imaging

**Figure 5:**
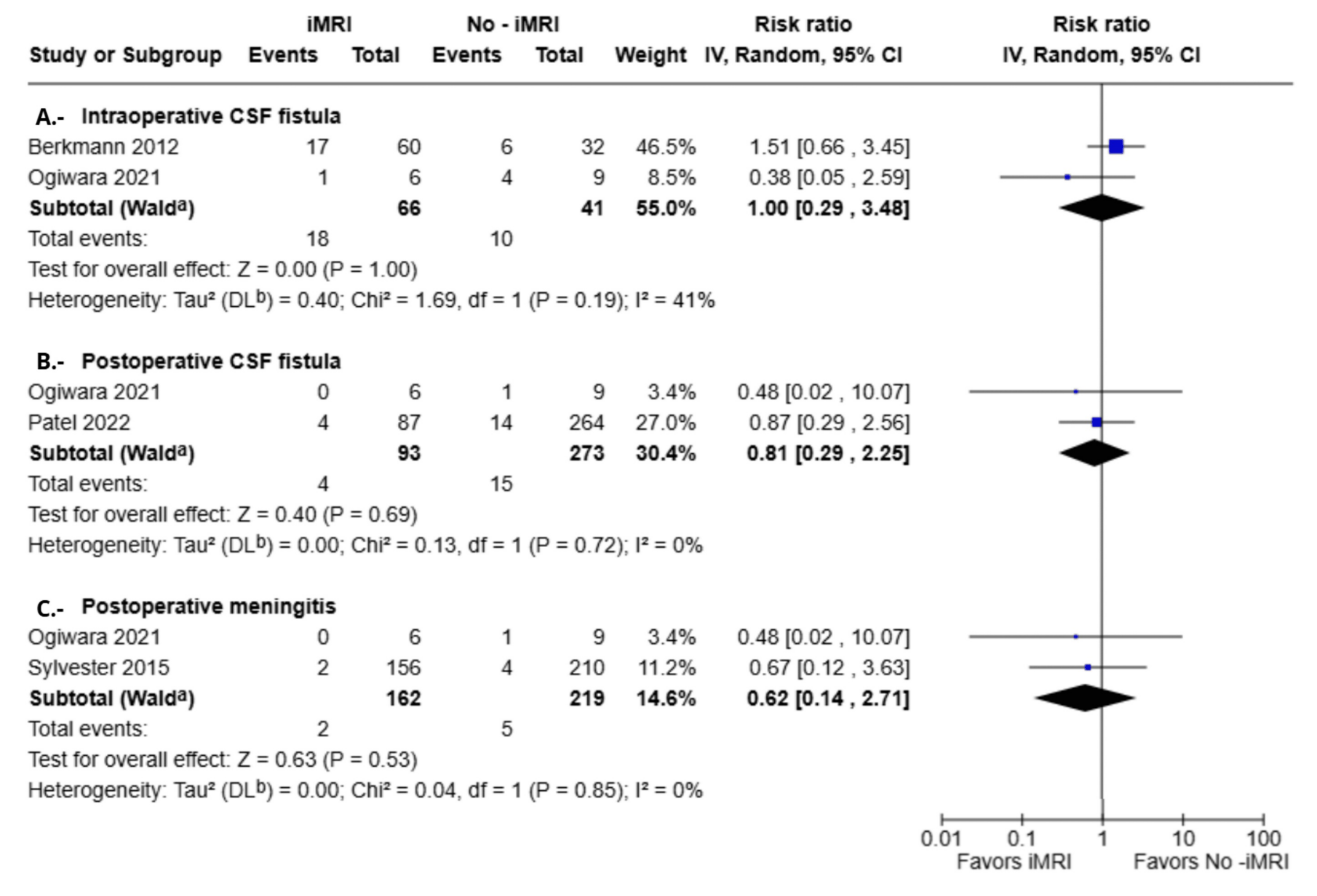
Forest plots of surgical complications comparing iMRI and non-iMRI groups Berkmann et al., 2012 [21], Ogiwara et al., 2021 [19], Patel et al., 2022 [18], Sylvester et al., 2015 [20] Forest plots illustrating the RR and 95% CI for intraoperative CSF fistula, postoperative CSF leaks, and meningitis in patients with and without iMRI during pituitary adenoma surgery. No statistically significant differences were found for any of the complications assessed (intraoperative CSF fistula RR = 1.00; 95% CI: 0.29-3.48; p = 1.00; postoperative CSF leaks RR = 0.81; 95% CI: 0.29- 2.25; p = 0.80; meningitis RR = 0.62; 95% CI: 0.14-2.71; p = 0.53). RR: risk ratio; CSF: cerebrospinal fluid; iMRI: intraoperative magnetic resonance imaging

In contrast, no statistically significant differences were observed between groups in terms of the need for additional surgical resection. The corresponding RR was 1.44 (95%CI: 0.03-73.65; p = 0.86), as depicted in Figure 4. Similarly, the incidence of intraoperative and postoperative complications, including CSF leaks and meningitis, did not differ significantly between groups. Specifically, the RR for intraoperative CSF leaks was 1.00 (95%CI: 0.29-3.48; p = 1.00), for postoperative CSF leaks was 0.81 (95%CI: 0.29-2.25; p = 0.69), and for meningitis was 0.62 (95% CI: 0.14-2.71; p = 0.53), as illustrated in Figure 5. Notably, none of the studies reported any deaths in either patient group.

Quality Assessment and Publication Bias

**Figure 6:**
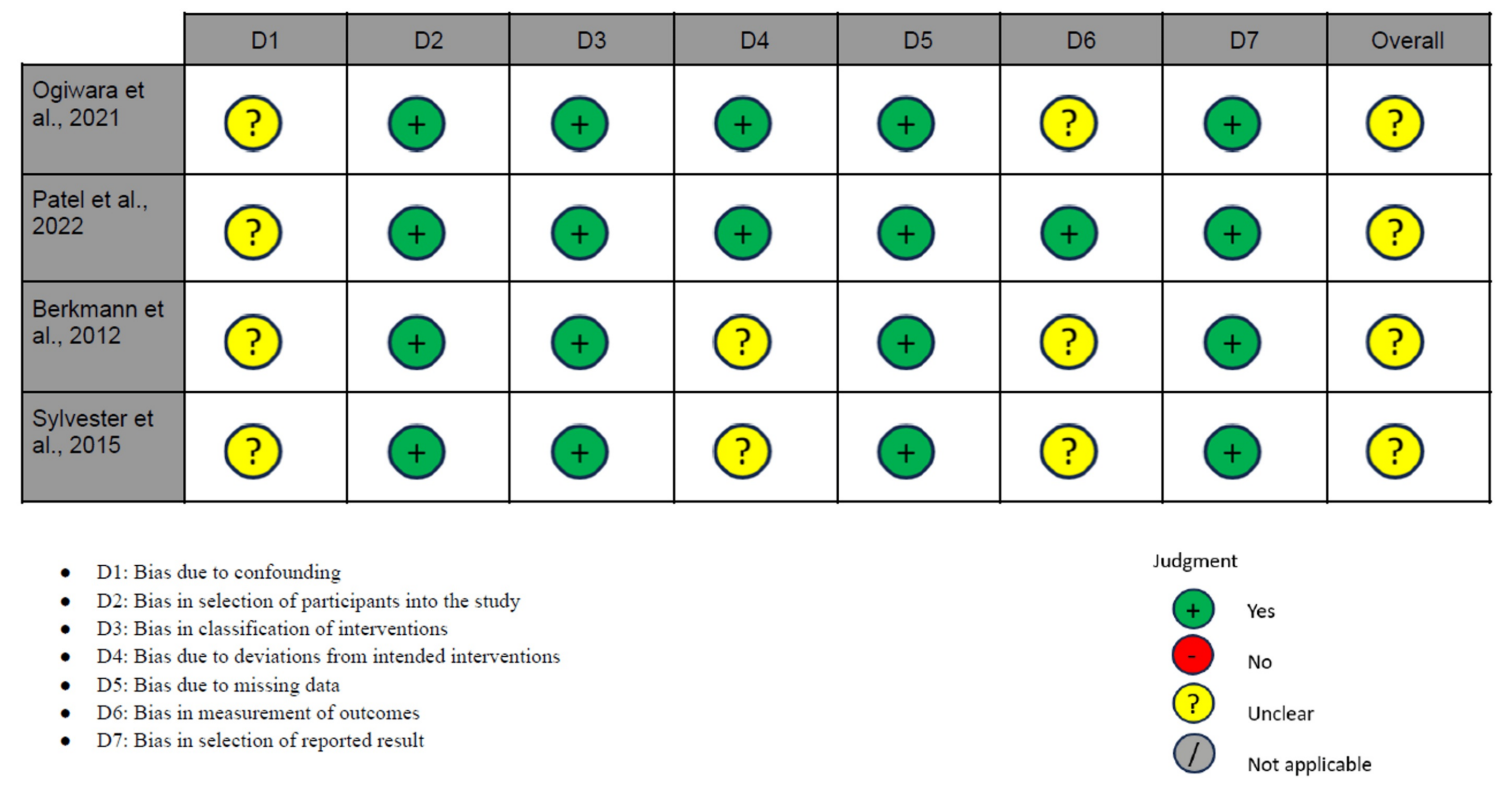
Risk of bias assessment using the ROBINS-I tool Ogiwara et al., 2021 [19], Patel et al., 2022 [18], Berkmann et al., 2012 [21], Sylvester et al., 2015 [20] Graphical summary of the risk of bias assessment using the ROBINS-I tool for non-randomized studies [9]. All included studies were judged to have a moderate overall risk of bias, primarily due to their retrospective design, potential uncontrolled confounding, and variability in outcome measurement and reporting. ROBINS-I: Risk of Bias in Nonrandomized Studies of Interventions

Methodological quality was assessed using the ROBINS-I tool [17], appropriate for non-randomized observational studies. All studies were classified as having a moderate risk of bias, mainly due to the retrospective nature of their design, the presence of potential uncontrolled confounding factors, and variability in outcome collection and reporting. A full summary of the quality assessment is provided in Figure 6.

**Figure 7:**
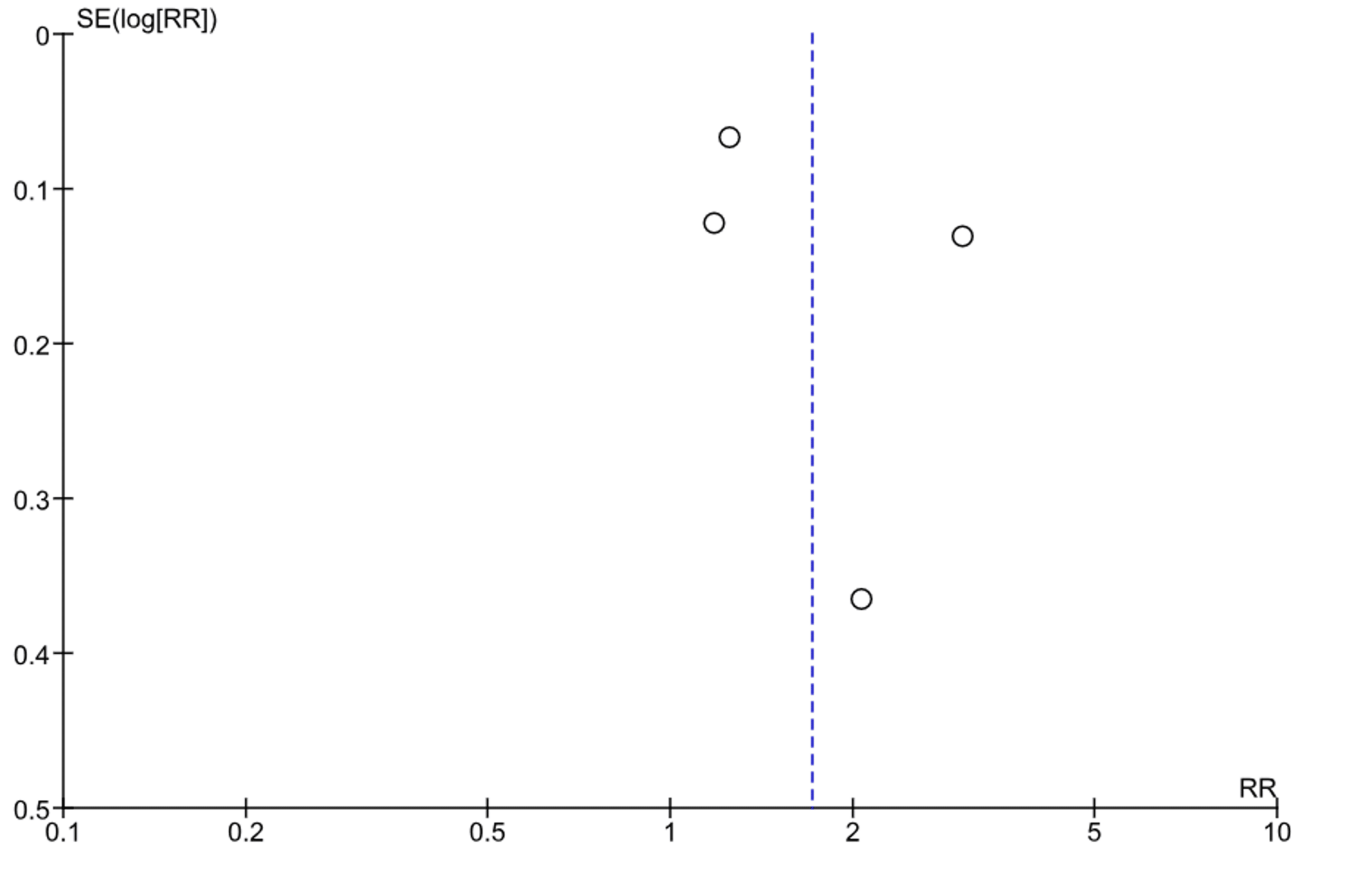
Funnel plot for publication bias assessment Studies included: Patel et al., 2022 [18], Ogiwara et al., 2021 [19], Sylvester et al., 2015 [20], Berkmann et al., 2012 [21] Funnel plot evaluating publication bias for the outcome of the final gross total resection. The relatively symmetrical distribution of studies around the pooled effect estimate suggests no major publication bias. However, the small number of included studies (n = 4) limits the statistical power of this analysis, and the presence of publication bias cannot be definitively excluded.

To explore publication bias, a funnel plot was constructed for the final GTR outcome (Figure 7). Visual inspection of the plot revealed a relatively symmetrical distribution around the overall effect estimate, with no clear signs of asymmetry. However, since only four studies were included, interpretation should be approached with caution, as the statistical power of this assessment is limited and publication bias cannot be entirely ruled out. A formal Egger’s test was not performed, as statistical tests for funnel plot asymmetry are generally not reliable when the number of studies is fewer than 10, according to Cochrane guidelines. To further ensure the reliability of our findings, a leave-one-out sensitivity analysis was conducted, confirming that the pooled results were robust and not disproportionately driven by any single study.

Discussion

The findings of this meta-analysis support the use of iMRI as an effective tool for optimizing GTR in PitNET surgery. These results are consistent with those reported by Patel et al. [18], who emphasized that iMRI assistance improves surgical outcomes in terms of achieving complete tumor resection. Beyond quantitative outcomes, the true value of this technology lies in its ability to provide real-time imaging, enabling surgeons to make strategic adjustments during the procedure, particularly in anatomically complex cases. 

In recent years, population-based and institutional studies have documented an increasing adoption of intraoperative MRI in transsphenoidal PitNET surgery, particularly in high-volume centers managing complex adenomas. Sharma et al. demonstrated a clear upward trend in iMRI utilization and highlighted its role in detecting residual tumor during the same operative session, albeit with increased operative time and healthcare resource utilization [2]. Early institutional experiences further support that iMRI facilitates intraoperative decision-making and refinement of resection strategy, especially during the learning curve of advanced endoscopic techniques [22]. These data suggest that the clinical value of iMRI extends beyond resection rates and reflects its role in surgical planning and intraoperative adaptability.

This advantage becomes even more evident in functional PitNETs, where incomplete resection may perpetuate hormonal disorders such as acromegaly, hypothyroidism, or hypogonadism. Studies such as that by Zhang et al. [11] reinforce this premise, demonstrating that more precise iMRI-guided resections can lead to improved morphological, endocrine, and functional outcomes in the postoperative period. 

The relevance of iMRI appears particularly pronounced in functional PitNETs, where even minimal residual tissue may compromise biochemical remission. In a consecutive single-center series of functional adenomas, Scherer et al. reported that iMRI-guided additional resection led to improved identification of tumor remnants and contributed to higher rates of endocrine control [23]. Similarly, in recurrent PitNETs, iMRI has demonstrated utility in identifying residual disease obscured by scar tissue or altered anatomy, potentially reducing early recurrence and the need for adjuvant therapies [24]. These findings reinforce the role of iMRI in hormonally active and previously operated tumors, where surgical margins are often less distinct.

However, it is crucial to distinguish between technical efficacy and safety profile. Despite the surgical visualization advantages offered by iMRI, the analyzed data did not demonstrate a significant reduction in complications such as CSF leaks or meningitis. This may be attributed to several factors: postoperative complications may be more influenced by variables such as the surgical closure technique, total operative time, the experience of the surgical team, or patient comorbidities rather than by the quality of intraoperative imaging itself. Thus, while iMRI enhances resection precision, it does not necessarily lead to a direct reduction in perioperative complications. In other words, "better visualization" does not inherently equate to "greater perioperative safety." 

Large multicenter and benchmark analyses indicate that perioperative complications in TSS are multifactorial and may be more closely associated with case complexity, patient characteristics, and surgical expertise than with adjunctive imaging alone. Drexler et al. established benchmark outcomes demonstrating that complication rates vary primarily with tumor invasiveness and institutional experience rather than specific intraoperative technologies [25]. Moreover, comparative studies between endoscopic and microscopic transsphenoidal approaches have shown that both techniques yield comparable safety profiles when appropriately selected [26]. Importantly, patient-related factors such as preoperative frailty have emerged as independent predictors of adverse events, further underscoring that iMRI cannot fully offset intrinsic surgical risk [27].

The clinical applicability of these findings must be carefully considered in context. Factors such as adenoma subtype, patient profile, and technological availability are key determinants. In this regard, Buchfelder et al. [28] underscore the importance of tailoring the surgical approach to the specific characteristics of each case. It is important to note, however, that the primary literature analyzed in this study did not provide a standardized or granular breakdown of histopathological subtypes according to current WHO lineages. While the distinction between functional and non-functional adenomas was maintained, the lack of more specific pathological data limits further sub-analysis. Therefore, the decision to incorporate iMRI into surgical protocols should not be based solely on its availability, but rather on clearly defined clinical criteria. 

Detailed analyses of tumor remnants after iMRI-assisted surgery suggest that its greatest benefit is observed in cases with invasive growth patterns, complex parasellar anatomy, or prior surgical intervention. Paľa et al. demonstrated that iMRI allows for more precise characterization of residual tumor tissue that may not be evident with standard visualization techniques [29]. Furthermore, prognostic models evaluating recurrence and progression emphasize that long-term outcomes depend on multiple tumor- and patient-related factors, including size, invasiveness, and biological behavior [30]. These data support a selective, patient-centered approach to iMRI integration rather than its routine use in all cases.

From a methodological standpoint, the included studies present notable limitations, including their retrospective design, heterogeneity in data collection, and the presence of potential uncontrolled confounding factors. Our leave-one-out sensitivity analysis revealed that the high heterogeneity observed in the primary outcome (I² = 93%) was primarily driven by the study by Sylvester et al. [20]. Upon exclusion of this study, heterogeneity decreased significantly to I² = 4%, while the benefit of iMRI remained statistically significant (RR = 1.25, p = 0.0003). This confirms that the observed clinical advantage of iMRI is robust across the dataset, despite the variability introduced by individual studies. 

Although the assessment of publication bias suggests a low risk of systematic distortion, the limited number of studies restricts the generalizability of the findings. Therefore, rather than overstating the observed benefits, these results should be regarded as a solid foundation for promoting critical and informed consideration of iMRI use in PitNET surgery.

Future research should focus on prospective, multicenter, controlled studies that evaluate not only resection rates but also functional outcomes such as quality of life, neuroendocrine recovery, return to daily activities, and short- and long-term cost-effectiveness analyses. Furthermore, the development of predictive models could be valuable in identifying which patients are most likely to benefit from the integration of iMRI into their surgical treatment.

## Conclusions

Based on the evidence synthesized in this meta-analysis, recent clinical and institutional evidence supports the role of iMRI as a valuable adjunct in TSS, particularly in centers managing complex, functional, or recurrent PitNETs. Large cohort analyses have demonstrated that iMRI contributes to improved intraoperative detection of residual tumor and may facilitate more informed surgical decision-making, although its use is associated with increased operative time and resource utilization. These findings underscore the importance of balancing potential clinical benefit with institutional capabilities and cost considerations.

Our meta-analysis identifies iMRI as an effective complementary intervention to enhance surgical precision and GTR rates, even when accounting for study heterogeneity through sensitivity analysis; however, while its impact on perioperative safety remains not clearly established, its strategic integration into selected protocols may translate into improved clinical and endocrinological outcomes, especially in functional and recurrent PitNETs where complete resection is closely linked to biochemical remission. Evidence from comparative studies suggests that iMRI-guided additional resection can improve the identification of tumor remnants that may otherwise go undetected, thereby potentially reducing the risk of persistent disease. However, perioperative safety remains influenced by multiple factors, including tumor complexity and surgical expertise, highlighting that adjunctive imaging alone does not eliminate inherent surgical risk. Current evidence supports its use as an adjunct rather than a substitute for surgical judgment and highlights the need for more robust studies to establish its definitive clinical and cost-effective value. Its strategic implementation represents a significant step toward more precise, personalized, and evidence-based neurosurgery.
